# Plant vigour at establishment and following defoliation are both associated with responses to drought in perennial ryegrass (*Lolium perenne* L.)

**DOI:** 10.1093/jxb/eru318

**Published:** 2014-08-06

**Authors:** Jean-Hugues B. Hatier, Marty J. Faville, Michael J. Hickey, John P. Koolaard, Jana Schmidt, Brandi-Lee Carey, Chris S. Jones

**Affiliations:** AgResearch Grasslands Research Centre, Private Bag 11008, Palmerston North 4442, New Zealand

**Keywords:** Drought, grass, *Lolium perenne*, moisture stress, pasture production, perennial ryegrass, quantitative trait locus (QTL), soil water content.

## Abstract

Periodic drought events present a significant and, with climate change, increasing constraint on temperate forage plants’ production. Consequently, improving plants’ adaptive response to abiotic stress is a key goal to ensure agricultural productivity in these regions. In this study we developed a new methodology, using both area-based comparison and soil water content measurements of individual non-irrigated and irrigated clones, to assess performance of perennial ryegrass (*Lolium perenne* L.) genotypes subjected to moisture stress in a simulated competitive environment. We applied this method to the evaluation of a full-sibling population from a pair cross between genotypes from a New Zealand cultivar and a Moroccan ecotype. Our hypothesis was that: (i) both leaf lamina regrowth after defoliation (LR) and plant vigour affect plant performance during drought and rehydration; and (ii) quantitative trait loci (QTLs) associated with plant performance under moisture stress could be identified. Differences amongst genotypes in dry matter (DM) production, early vigour at establishment, leaf elongation rate and LR were measured. LR explained most of the variation in DM production during exposure to moisture deficit and rehydration followed by plant vigour, indicated by initial DM production in both treatments and subsequent measures of DM production of irrigated clones. We identified two main QTL regions associated with DM production and LR, both during drought exposure and rehydration. Further research focused on these regions should improve our understanding of the genetic control of drought response in this forage crop and potentially other grass species with significant synteny, and support improvement in performance through molecular breeding approaches.

## Highlight text

We have developed a new methodology to assess individual perennial ryegrass plant performance under moisture stress and identified QTLs associated with improved performance during drought in this important forage species.

## Introduction

Perennial ryegrass (*Lolium perenne* L.) is widely used in grazing systems across the temperate world (Casler and Kallenbach, 2007). However, the growth and development of this grass can be significantly constrained by periodic drought events (Barker *et al.*, 1985; Liu and Jiang, 2010) and its distribution in temperate environments is limited to areas with a minimum of 700mm annual rainfall (Moore, 1969). Consequently, improving plant persistence, including adaptive response to abiotic and biotic stress factors, is a key objective in perennial ryegrass breeding programmes (Woodfield and Easton, 2004; Yu *et al.*, 2013). Local climate can also influence the development of plants, with plant growth curves often specific to ecogeographical regions (Radcliffe and Lynch, 1974; Rickard and Radcliffe, 1976). Consequently, for a robust assessment of genotype performance during drought, defined here as ‘the capability of the plant to maintain dry matter (DM) yield production during drought’, it is crucial that the drought treatments imposed in experimental systems are similar to the stresses that occur naturally, both in intensity, duration, and timing of initiation of drought (Da Silveira Pinheiro, 2003), and that factors such as ambient temperature and photoperiod are considered alongside drought intensity.

To cope with the harmful effects of moisture deficit stress, plants have developed a variety of adaptive mechanisms, including drought tolerance (Ludlow, 1989; Turner, 1986), dehydration escape (Whalley, 1973), dehydration avoidance (Sinclair and Purcell, 2005), and enhanced passive processes such as hydraulic redistribution (Howard *et al.*, 2009). In addition, for perennial grasses to be compatible with their role within agricultural systems, these plants must often not only tolerate and persist after drought but also survive sudden removal, through defoliation by livestock, of their vegetative organs that control water loss through stomatal regulation under moderate stress conditions (Carter, 1978; Acharya *et al.*, 2004). Therefore, it is important to understand a plant’s response to the combined effects of drought and defoliation stressors when considering mechanisms of persistence in these grasses (Boschma *et al.*, 2003).

Many studies have been performed on drought resistance of forage species and these have provided a foundation of relevant information for the analysis of plant performance under moisture stress (Wedderburn *et al.*, 1992; Karatassiou and Noitsakis, 2010; Liu and Jiang, 2010; Gallego-Giraldo *et al.*, 2011; Holloway-Phillips and Brodribb, 2011; Kane, 2011; Lelièvre *et al.*, 2011; Man *et al.*, 2011; Pecetti *et al.*, 2011). This study aims to utilize the knowledge generated in these studies and bring its relevance into a sustainable farming system environment where forage-based feed, which underpins animal production, directly depends on plant performance and persistence. Using a semi-controlled environment to regulate soil moisture content at the individual plant level, our primary objective was to test the hypothesis that both leaf lamina regrowth after defoliation (LR) by mechanical trimming and plant vigour, defined here either by initial DM production at establishment or by DM production when water resources are not limiting at each time point, affect plant performance both during drought and subsequent rehydration.

Our second objective was to identify quantitative trait loci (QTLs) that support DM production in perennial ryegrass during drought and rehydration of plants following the alleviation of drought stress. The identification of genomic regions that influence DM production in response to drought will improve our understanding of the genetic mechanisms of dehydration tolerance and may be exploited through marker-assisted selection (MAS) to assist plant breeders to improve yield performance of perennial ryegrass cultivars in environments where periods of soil moisture deficit are experienced. The development of MAS to support breeding for this trait would reduce dependence on complicated, costly, and time consuming phenotypic screening.

## Materials and methods

### Plant material

Perennial ryegrass mapping population *RM4* is a random sample of the full-sibling F_1_ progeny from a pair cross between a genotype from the New Zealand cultivar ‘Grasslands Samson’ and a genotype from the semi-arid North African state of Morocco. The maternal ‘Grasslands Samson’ parent is the same genotype used to develop a previously described mapping population (‘I × S’; Sartie *et al.*, 2011). The Moroccan genotype was sourced from an accession (PI 598854) obtained from the National Plant Germplasm System of the USDA-ARS. Anecdotal evidence suggests that genotypes from this accession should perform well under moisture stress. The *RM4* population was assessed in a drought experiment and used to construct a genetic linkage map with the aim of detecting QTLs associated with drought response and drought adaptation traits. Plants were vernalized during the southern hemisphere winter (July–August).

### Experimental design in Palmerston North

A total of 147 genotypes, assessed with simple sequence repeat (SSR) markers to confirm their identity as members of the *RM4* mapping population, were clonally propagated to make plants available for two treatments (non-irrigated and irrigated) with three clonal replicates within each treatment. All the plants were transplanted on 7 October 2009 into soil in an automated rainout shelter situated at the AgResearch Grasslands site in Palmerston North, New Zealand. The trial was established as a repeated row–column design which was optimized to avoid clonal replicates from being present twice in the same row or column. Trenches, 0.5 m deep, had been dug between blocks to avoid any water leakage and each experimental block was surrounded by a single buffer row of ryegrass plants. Irrigation was the only source of moisture applied throughout the experiment as the rainout shelter completely covered the experimental area automatically when precipitation was detected. The use of the rainout shelter allowed precise control over environmental factors required for reproducible QTL mapping studies (Bruce *et al.*, 2002; Mitchell *et al.*, 2013).

To establish the experiment, small plants, consisting of ~20 tillers with laminae trimmed to 20mm above the ligule, were transplanted into bare soil in a grid pattern at a spacing of 350mm. A 15mm high ring cut from a piece of 50mm diameter PVC pipe was then fully inserted into the soil surrounding each plant to limit its lateral expansion above ground. Limiting expansion with the pipe facilitates the assessment of plant performance per unit area which enables the comparison of individual genotype performance in an environment closely resembling that experienced in a competitive sward. A drip irrigation system was installed in both treatment areas. Soil nutrient status was adjusted following a soil test to meet the recommendations of Roberts and Morton (2004).

### Climatic data

**Fig. 1.**
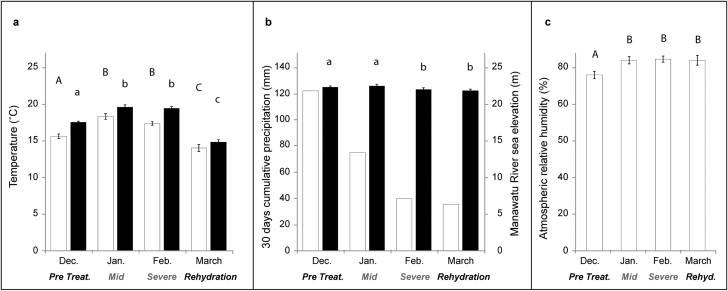
(a) Change in average air (open bars) and 20cm depth soil (filled bars) temperatures. (b) Total monthly precipitation (open bars) and average elevation of the Manawatu River near the experimental site (filled bars) modified from the hydrometric data as supplied by Horizons Regional Council, Palmerston North. (c) Average relative humidity (RH) during the course of the Palmerston North experiment. Means ± s.e.; bars with different letters above are significantly different at *P* < 0.05.

To confirm that our experiment was performed under climatic conditions normally experienced while moisture deficit naturally occurs, weather data were collected from the Palmerston North Ews NIWA weather station located adjacent to the experimental site (Agent Number 21963, Network Number EO536D, Latitude 40.38195 S, Longitude 175.60915 E; Fig. 1).

Data on the water level of the Manawatu River at the Teachers College monitoring station within 2 km of the experimental site were provided by the Horizons Regional Council, Palmerston North (http://www.horizons.govt.nz/managing-environment/resource-management/water/river-heights-and-rainfall/), hydrometric data as supplied on 10 December 2010. These data provide an indication of the water table near our experimental site.

### Drought and defoliation treatments

**Fig. 2.**
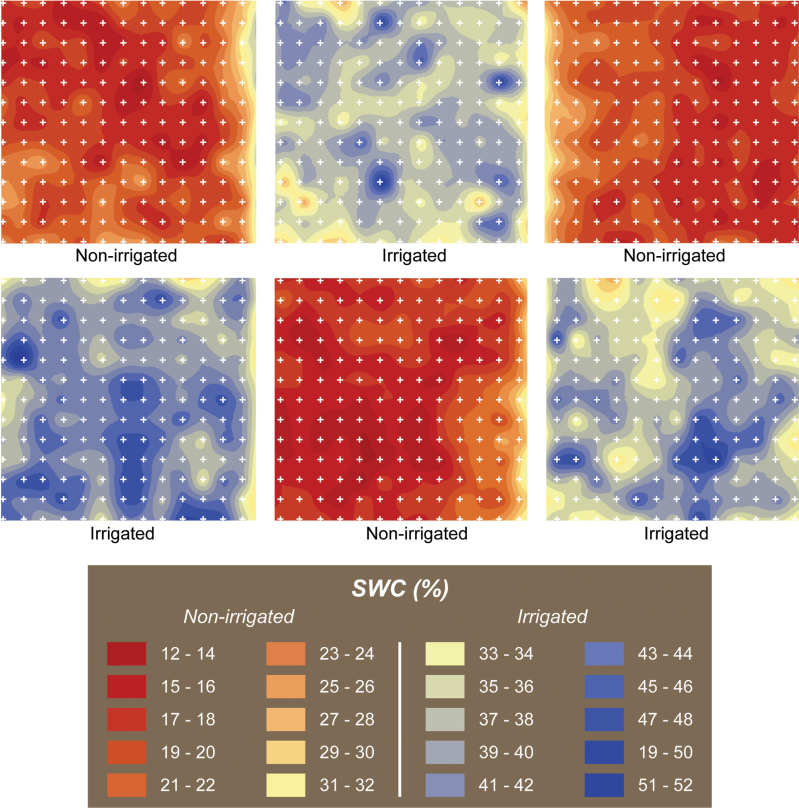
Variation in volumetric SWC (%) across the experimental sites 63 days after the drought treatment was applied at Palmerston North. Each white cross represents a measurement location.

Plants were allowed to expand until the majority of the clones had colonized the entire soil surface delimited by the pipe ring. During this period, plants were irrigated every second day to ensure full turgidity. Initial measurement of dry matter (DM-Pre) was carried out on 12 January 2010 by cutting with electric shears to 50mm above ground level. At this time, half of the plants continued to receive the same watering treatment (control) and irrigation was withheld from the other half for 63 days, with the aim of having volumetric soil water (SWC) content representing severe drought conditions in the New Zealand environment (Fig. 2). Further DM measurements were taken, as described above, after 24 days (DM-Mid, mid drought) and again after 63 days (DM-Severe, severe drought) from the initiation of the non-irrigated treatment. Irrigation was then supplied to all plants which were grown for a further 32 days before a final cut (DM-Post, rehydration; Table 1). All foliage samples were dried at 80°C overnight and weighed to assess DM production.

**Table 1.** List of traits measured in perennial ryegrass mapping population *RM4* at different stages of the experiment

Trait	Date	Phase (days)	Plot	Irrigation	Symbol
Shoot DM	13/01/10	Pre treatment (32)	Control	On	DM-Pre
			Drought	On	
	5/02/10	Mid drought (24)	Control	On	DM-Mid_irr
			Drought	Off	DM-Mid_non
	16/03/10	Severe drought (39)	Control	On	DM-Severe_irr
			Drought	Off	DM- Severe_non
	15/04/10	Post Rehydration (32)	Control	On	DM-Post_irr
			Drought	On	DM-Post_non
Early vigour at establishment	13/01/10	Pre drought	Both	–	–
LER	12/02/10	Mid drought	Control	On	LER_irr
			Drought	Off	LER_non
SWC	1/03/10	Severe drought	Both	–	–
LR	01/03/10	Severe drought	Control	On	LR_irr
			Drought	Off	LR_non

### Endophyte status

In order to eliminate any potential confounding effect associated with the presence of the fungal endophyte *Neotyphodium lolii*, which has been shown to confer enhanced plant performance under drought conditions (Elmi and West, 1995; Elbersen and West, 1996; Cheplick *et al.*, 2000; Hesse *et al.*, 2003), all genotypes were confirmed to be endophyte free by immunoblotting using two tillers per plant (Hahn *et al.*, 2003).

### Plant score (early vigour at establishment)

At the end of the establishment period, plants were scored for ring area colonization success as follows: 0, dead; 1, <3 tillers alive; 2, plant green but full area not yet covered; 3, full area covered but below average production; 4, average production; 5, above average production; 6, plant exceptionally vigorous.

### Soil water content

Volumetric soil water content (SWC) at each plant position was measured, using a 200mm CS620-Hydrosense digital Time-domain reflectometer (Campbell Scientific Inc), during each harvest. The 200mm rod was chosen for this experiment because more than 70% of perennial ryegrass roots occur within that horizon in the field (Jacques, 1943; Reid and Crush, 2013).

### Leaf elongation rate at mid drought

Two tillers per plant were randomly selected and tagged with coloured labels for leaf elongation rate (LER) measurements. Leaf length measurements, measuring from the top of each emerging leaf lamina to the ligule of the next oldest leaf, were taken at the time of tagging and again after two days (Norris and Thomas, 1982). An average of the LER measurements collected from the two tillers was calculated for each plant.

### LR

Plants were scored for their potential to survive trimming at the end of the establishment period and 24 days later at the DM-Mid sampling stage. Scores on a scale of 0–3 were attributed to each individual plant as follows: 0, plants that died after trimming, confirmed by the fact that they did not produce any green material two months after rehydration; 1, plants with a few leaf laminae growing; 2, plants that had more than half their leaf laminae growing; and 3, plants where trimming did not appear to influence leaf lamina regrowth.

### Supplementary experiment in Lincoln

To test the reproducibility of the results of the main experiment in a second environment, 40 genotypes from the *RM4* mapping population, selected to represent high- and low-performing groups of plants in equal proportions, were grown in a second experiment. This was initiated in September 2010, employing an automated rainout shelter situated in Lincoln, New Zealand, following the protocol described above. The high-performing group was composed of 20 genotypes that performed well based on DM production under severe moisture deficit and after rehydration in the 2009 experiment. The low-performing group was composed of 20 genotypes with significantly reduced performance when grown under severe moisture deficit and after rehydration but which did not differ from the high performing group, in terms of DM production, in the control treatment.

All the plants were transplanted into soil in a repeated row–column design, with four replicates per water treatment, following the method described above. Net rate of photosynthesis (Pn), relative water content (RWC), and carbon isotopic abundance (δ^13^C) were estimated at the end of the establishment period, during severe drought, and after rehydration. Pn measurements were made using a portable photosynthesis system (Li6400, LiCor Inc., Lincoln, NE, USA) fitted with standard 2×3cm leaf chamber, leaf thermocouple, and a blue-red LED light source. Each entry was measured, at 1500 µmol m^–2^ s^–1^ PAR (field growing conditions), between 11.00 and 16.00 hours, over a three-day time period. Block temperature was held at 20°C, stomata ratio was set at 1.6, and the vapour pressure deficit was between 0.6 and 0.9 kPa.

RWC was determined using the procedure described by Bayat (2009). Briefly, four first fully expanded leaves per plant were randomly collected and their fresh weight (FW) measured. Samples were then placed in the dark for 24h at 5°C in tubes filled with distilled water to achieve full turgidity and turgid weight (TW) was measured. Leaves were then oven-dried for 48h at 70°C and their DW was measured. RWC was estimated as follows: (FW–DW)/(TW–DW).

The δ^13^C analysis was carried out on a fully automated Europa Scientific 20/20 isotope analyser located at The University of Waikato Stable Isotope Unit. Samples were combusted, and the resulting gases separated by gas chromatography and then analysed by continuous-flow mass spectrometry.

### Statistical analysis

Genotype means were calculated using a mixed effects model which included the block as a random effect, and genotype as a fixed effect. Row and column effects were included in the model but they explained only a small amount of the random variation so the final model was re-fitted without those factors. The edge effect was assessed using an indicator variable identifying those plants on the edge and those towards the middle, but this effect was also negligible so the factor was omitted from the final model. Each treatment differed substantially with respect to the variance and distribution of the measured characteristics. A square-root transformation was required for most measurements, especially those that involved DM, to suitably stabilize the variance. Plant early vigour scores were used as covariates in the statistical analysis to allow a full comparison between genotypes. Even though analyses using SWC as a covariate gave similar results to those without it, the use of SWC as a covariate was considered to be less accurate from a physiological point of view as the relationship between SWC and soil water potential is not linear (Clapp and Hornberger, 1978). Therefore we decided to present the statistical analysis results obtained without using SWC as a covariate.

The means of each genotype were obtained, together with the average standard error of the difference between the means (SED). Regression analyses were used to estimate the partial contribution of the DM-Pre and the control DM of the irrigated clones, LR, and LER, in explaining the variation in DM production at each time point.

### Genetic linkage map development

DNA was isolated from leaf tissue of 147 progeny from *RM4* and the ‘Grasslands Samson’ parent using the FastDNA kit procedure as per the manufacturer’s instructions (QBiogene, Carlsbad, CA, USA). A total of 102 SSR markers were used to assay the ‘Samson’ parent and individuals from *RM4*, including 78 perennial ryegrass SSR markers derived from expressed sequence tags (ESTs; previx ‘pps’) (Faville *et al.*, 2004), 19 developed from GeneThresher^®^ sequences (prefix ‘rv’) (Gill *et al.*, 2006) and five from a reference set of markers (Bach Jensen *et al.*, 2005). For all SSR markers, PCR and capillary electrophoresis of PCR products were conducted according to Sartie *et al.* (2011). Electropherograms were analysed and fragments sized using GeneMarker v1.75 (SoftGenetics, LLC, PA, USA). DNA could not be obtained from the Moroccan parent as the plant had died and been discarded prior to commencing the experiment. Markers selected for genotyping were all known to be heterozygous in the ‘Grasslands Samson’ parent following its use in a related population (Sartie *et al.*, 2011). At each SSR locus the genotype of the Moroccan parent was inferred based on observations of the ‘Grasslands Samson’ parent allele configuration and those of the segregating F_1_ progeny. More precisely, at a given marker, any segregating allele(s) observed in the F_1_ progeny that fitted an expected segregation ratio for a two-way pseudo-testcross (Grattapaglia and Sederoff, 1994), but which was not present in the Samson parental genotype, was assigned to the Moroccan parent. Alleles present in both parental genotypes were similarly inferred.

This process enabled the construction of genetic linkage maps for both the Samson and Moroccan parental genotypes and a two-way pseudo-testcross analysis was used to construct an SSR-based genetic linkage map in *RM4*, using the cross pollination (CP) population module in JoinMap® 3.0 (Van Ooijen and Voorrips, 2001). Individual parental maps were constructed first and checked for conservation of marker locus order. A consensus map was then estimated, based on meioses in both parental genotypes. Marker grouping occurred at a logarithm-of-odds (LOD) score of 10.0. Ordering of markers within groups was constrained by thresholds of LOD > 2.0, recombination frequency < 0.40, and jump in goodness-of-fit < 5.0. Map distances in centimorgans (cM) were calculated from recombination frequencies using the Kosambi mapping function (Kosambi, 1944). Linkage group (LG) assignments are consistent with the International *Lolium* Genome Initiative (ILGI) nomenclature and correspond to the homoeologous groups of the *Triticeae* cereals (Jones *et al.*, 2002*a*; Jones *et al.*, 2002*b*).

For mapped perennial ryegrass ESTs, sequence alignment using the Basic Local Alignment Search Tool, BLASTN (Altschul *et al.*, 1990; Altschul *et al.*, 1997) or the BLAST-like Alignment Tool, BLAT (Kent, 2002), (threshold values of <E^–15^; SID > 85% over >100bp) were used to estimate similarity with homologous sequences in the TIGR rice pseudomolecule assembly of the rice genome hosted at www.gramene.org.

### QTL analysis

QTL analysis was conducted using the *RM4* consensus genetic linkage map and phenotypic data from the 147 F_1_ genotypes evaluated in the Palmerston North drought response experiment. The transformed mean values for each trait were used first to conduct interval mapping (IM), implemented using MapQTL® 4.0 software (Van Ooijen *et al.*, 2002). Estimated positions and the magnitude of QTLs were refined using the multiple QTL mapping (MQM) module, following the procedure detailed by Sartie *et al.* (2011) and modified by Khaembah *et al.* (2013). Permutation testing (n = 2000) for each trait established LOD thresholds for QTL declaration at a linkage group- or genome-wide significance of *P* < 0.05 (Churchill and Doerge, 1994). QTL positions were defined by 1- and 2-LOD confidence intervals. A second criterion for QTL acceptance was the presence of a significant (*P* < 0.01) Kruskal-Wallis single marker test within the QTL 2-LOD confidence interval (data not shown).

Phenotype means for four different CP population structure QTL allele configurations (ac, ad, bc, and bd), calculated using MapQTL 4.0, were used to estimate additive allelic effects at the detected QTL following the model of Knott *et al.* (1997) as described previously (Sewell *et al.*, 2002; Sartie *et al.*, 2011).

## Results

### Climatic data

Weather monitoring data showed that the average temperatures in soil at 20cm depth and in ambient air followed the same patterns during the course of the experiment (Fig. 1a) with both temperatures being on average about 30% higher during the two months of drought (January and February, in the southern hemisphere summer) than during the month of rehydration. The amount of cumulative precipitation decreased from December onward to reach a minimum of 36mm in March (Fig. 1b). Lowering of the water table was indicated by lower flow rates in the major river located near the trial site which was consistent with typical summer drought conditions (Fig. 1b). Relative humidity (RH) was on average 8% lower in December than during the following months and did not differ among the three months that the experiment was carried out (Fig. 1c).

### Trial homogeneity at starting point

There were no significant (*P* > 0.05) differences observed for plant performance at the end of the establishment period between plants transplanted in the ‘irrigated’ and ‘non-irrigated’ blocks (data not shown). Overall, plant score, volumetric SWC, LER, and LR average values were 3.96±0.32, 38.30% ± 3.54%, 10.38±2.65mm day^–1^, and 3.8±0.2, respectively.

### SWC

At the time of severe drought, 63 days after the non-irrigated treatment was applied, volumetric SWC varied from 12% to 32% in the non-irrigated plots and from 33% to 52% in the irrigated plots (Fig. 2). There was no evidence of a genotype effect on SWC (Tables 2 and 3). Thus, in order to ensure segregation between, and homogeneity within, treatments, individual plants with SWC greater than 23% in the non-irrigated plots (54 plants) and those with SWC lower than 33% in the irrigated plots (41 plants) were excluded from the analysis.

**Table 2.** Wald tests for fixed effects on genotypes for volumetric SWC, LR, and LER for perennial ryegrass plants subjected to drought in the Palmerston North experiment

Fixed term	Wald statistic	d.f.	Wald/d.f.	*P* ^a^	
Genotype _**SWC**_	139.23	137	1.02	0.431	ns
Genotype _**LR**_	167.96	137	1.23	0.037	*
Genotype _**LER**_	143.08	135	1.06	0.301	ns

^a^ Significance levels: *P < 0.1; ns, not significant.

### LER

There was some evidence of significant differences in means among genotypes for defoliation response as estimated by leaf lamina regrowth after defoliation scoring for both the non-irrigated and irrigated treatments in this experiment (Tables 2 and 3). No evidence of a genotype effect on LER was found in the non-irrigated plots (Table 2). However, strong evidence of a genotype effect on LER was found in the irrigated plots (Table 3).

**Table 3.** Wald tests for fixed effects on genotypes for volumetric SWC, LR, and LER for perennial ryegrass plants subjected to the irrigated treatment in the Palmerston North experiment

Fixed term	Wald statistic	d.f.	Wald/d.f.	*P* ^a^	
Genotype _**SWC**_	145.56	143	1.02	0.425	ns
Genotype _**LR**_	186.32	143	1.30	0.009	*
Genotype _**LER**_	278.49	143	1.95	<0.001	***

^a^ Significance levels: *P < 0.1, ***P < 0.01, ns, not significant.

### Multiple regression analysis of plant performance

**Fig. 3.**
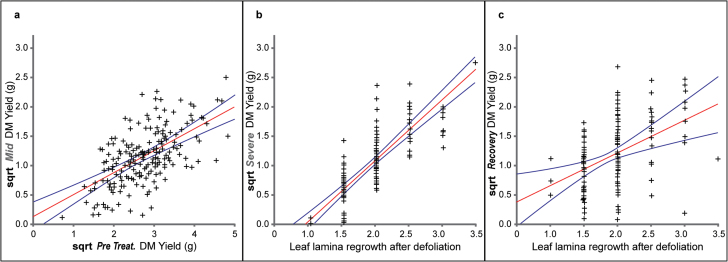
(a) Terms showing the strongest relationship with square root (sqrt) transformed DM production at (a) mid drought, (b) severe drought, and (c) after rehydration. The model explained 45% of the variation at mid drought (*P* < 0.001) with both DM-Pre and LR showing the strongest relationship, 65% at severe drought (*P* < 0.001), and 55% after recovery (*P* < 0.001) with leaf lamina regrowth after defoliation showing the strongest relationship for the two last time points. The two external curves in each graph represent the relationship with 95% confidence limits between fitted and observed data. This figure is available in colour at *JXB* online.

45% of the variation in DM production detected in the mid-drought treatment in January was explained by a model which incorporated DM-Pre, LR, and DM-Mid irrigated clones and LER. LR and DM-Pre explained most of the variation, with 24% associated with LR and 19% with DM-Pre (Fig. 3). In the severe drought treatment in February, the model explained 65% of the variation detected in DM production during drought, with LR explaining the majority (57%) of the variation detected for all of the terms and with DM production from the irrigated clones representing 8% of the variation. After rehydration, in March, the model explained 55% of the variation in DM production with LR, DM production of the irrigated clones in March, and DM production during severe drought contributing 38%, 12%, and 5% respectively. All of these terms have a *P* < 0.001.

### Detailed analysis in a second environment

The DM production results for the low- and high-performing plant groups in the Lincoln experiment were consistent with performance in the Palmerston North experiment (data not shown). There was some evidence of significant differences in means among genotypes for Pn and RWC and strong evidence of a genotype effect on δ^13^C in the non-irrigated plots (Table 4). DM production was negatively correlated with carbon isotopic abundance and positively correlated with RWC and Pn with R^2^ values of –32%, 31%, and 21%, respectively at the severe drought stage and –52% for δ^13^C and 36% for Pn after rehydration.

**Table 4.** Wald tests for fixed effects on genotypes for Pn, δ^13^C, and RWC for perennial ryegrass plants subjected to moisture stress in the Lincoln experiment

Fixed term	Wald statistic	d.f.	Wald/d.f.	*P* ^a^	
Genotype _***Pn***_	54.50	32	1.70	0.030	*
Genotype _**δ13C**_	409.07	30	13.68	<0.001	***
Genotype _**RWC**_	65.76	32	2.05	0.010	*

^a^ Significance levels: *P < 0.1, ***P < 0.01

### QTL analysis

**Fig. 4.**
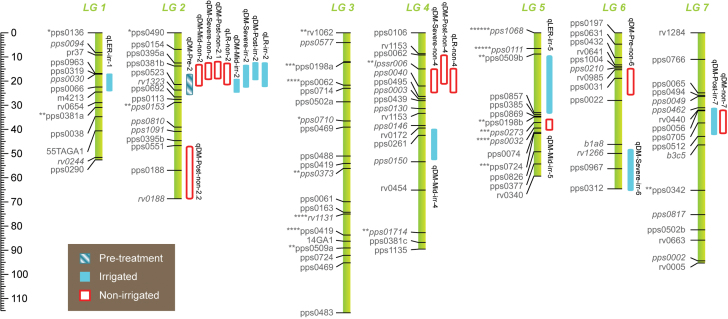
The genetic linkage map estimated for perennial ryegrass population *RM4*, showing seven linkage groups (LG1–LG7) and QTLs discovered for traits measured in a drought response experiment: DM-Pre (dry matter, pre-treatment), DM-Mid (24 days’ treatment), DM-Severe (63 days’ treatment), DM-Post (95 days’ treatment). Length of each LG is indicated by the centimorgan (cM) scale at the left of the figure. SSR marker loci are shown on the left of each LG and segregation distortion is indicated next to marker names (significance levels: *P < 0.1, **P < 0.05, ***P < 0.01, ****P < 0.005, *****P < 0.001, ******P < 0.0005). Italicized marker names represent bridging loci. QTLs are indicated by blocks on the right of LGs (2-LOD support interval). This figure is available in colour at *JXB* online.

Using genotypic data from 102 SSR markers, a genetic linkage map, spanning 564 cM of the perennial ryegrass genome, was constructed (Table 5; Fig. 4). The map consists of 111 loci, with eight SSR markers detecting multiple loci. The map has moderate marker saturation with a mean gap of 4.9 cM between loci and sub-optimal marker coverage in some regions due to seven moderately large inter-locus gaps (Table 5). Distorted segregation ratios were evident for 21% of the mapped loci at *P ≤* 0.05 (Fig. 4), with the majority of the distorted markers concentrated in discrete regions on LG3 and LG5.

**Table 5.** Features of the biparental consensus genetic linkage map developed for QTL discovery in the *RM4* mapping population of perennial ryegrass^a^

Map feature	LG1	LG2	LG3	LG4	LG5	LG6	LG7	Total
Number of loci	14	15	20	18	14	13	17	111
Number of bridging loci	3	5	4	7	4	3	5	31
Length (cM)	53	69	116	89	59	65	95	546
Mean locus density (cM locus^–1^)	3.8	4.6	5.8	4.9	4.2	5.0	5.6	4.9
No. interlocus gaps >15 cM	0	0	2	1	1	1	2	7

^a^ Bridging loci are those used to join two parental maps to form the consensus map.

Population mean values and ranges for traits evaluated in the QTL analysis are presented in Table 6. For all but two traits, LR under both irrigated and non-irrigated treatments, square root transformed data were used in the QTL analysis to restore a normal distribution. Phenotypic values for the ‘Grasslands Samson’ parent tended towards the mid-range of the F_1_ progeny distributions across all traits. This may be indicative of transgressive segregation in the progeny but this cannot be substantiated without phenotypic data from the Moroccan parent, which was absent from the experiment.

**Table 6.** Parent and progeny trait value means and ranges,and LSD and normality testing results in the F_1_ mapping population RM4^a^

Trait		Phase	Plot	Parent ‘S’ mean	F_1_ progeny mean	F_1_ progeny range	LSD (*P* < 0.05)	Normality (*P* value)^b^	Normality post- transformation^c^ (*P* value)
*Shoot DM*	DM-Pre	Pre	Both	4.8	7.55	2.36–15.73	2.24	<0.001	0.224
	DM-Mid	Mid	Control	1.1	1.73	0.17–4.30	0.87	0.003	0.982
			Drought	1.1	1.25	0.03–4.79	1.32	<0.001	0.701
	DM- Severe	Severe	Control	4.7	3.53	0.45–9.39	1.32	0.044	0.437
			Drought	1.6	1.38	0.00–4.24	1.62	<0.001	0.501
	DM-Post	Rehydration	Control	5.7	5.08	0.00–13.80	1.67	<0.001	0.727
			Drought	2.1	1.79	0.00–5.12	2.06	<0.001	0.135
LER		Severe	Control	3.2	3.23	1.68–4.50	1.31	0.136	–
			Drought	1.7	1.81	1.07–2.71	1.20	0.209	–
LR		Severe	Control	12.0	7.55	1.25–18.26	2.00	0.010	0.679
			Drought	3.7	4.95	0.98–3.54	2.27	<0.001	0.602

^a^ Parent data was collected for the maternal parent ‘Grasslands Samson’ only. ^b^ Shapiro-Wilk test for normality (*P* < 0.05 indicates data from a non-normal distribution). ^c^ Square root transformation for all traits except LR irrigated and LR drought for which no transformation was needed.

Across all traits, 20 significant QTLs were detected by MQM (Fig. 4, Table 7). Individual QTLs accounted for between 8 and 18% (mean = 12.2%) of total phenotypic variance (PV) for the trait. Total PV explained by all of the QTLs detected for a trait ranged from 13 to 46% (mean = 24.5%). The QTLs were located at 10 discrete genomic positions and one or more QTLs were detected on all LGs except for LG3, with notable clustering at the proximal ends of LG2 and LG4, and the central region of LG7 (Fig. 4).

**Table 7.** QTLs identified by IM and MQM for DM, post-drought LR, and LER under control and drought treatments in the *RM4* perennial ryegrass population^a,b^

Trait	Phase	Plot	QTL	LG	LOD Threshold		LOD score		PV (%)	2-LOD peak (cM)	Closest marker	S	M
LG wide	Genome wide	IM	MQM
Shoot DM	Pre	Both	qDM-Pre-2	2	2.8	3.7	2.9	4.1	11.1	18–26	rv1323	–0.40	0.56
			qDM-Pre-6	6	2.8	3.7	3.1	4.2	12.1	15–26	pps0031	–0.68	–0.22
	Mid	Control	qDM-Mid_irr-2	2	2.7	3.8	3.9	5.9	13.7	21–25	rv1323	–0.14	0.45
			qDM-Mid_irr-4	4	2.9	3.8	3.6	5.8	16.6	40–53	pps0261	–0.25	0.38
			qDM-Mid_irr-6	5	2.7	3.8	–	3.8	8.1	36–40	pps0032	–0.27	–0.28
		Drought	qDM-Mid_non-2	2	2.6	3.7	2.6	5.9	16.9	13–22	pps0523	–0.20	0.68
	Severe	Control	qDM-Severe_irr-2	2	2.7	3.8	–	4.3	11.8	13–23	pps0523	0.01	0.73
			qDM- Severe_irr-6	6	2.7	3.8	3.0	3.8	11.0	48–65	pps0967	0.01	–0.24
		Drought	qDM- Severe_non-2	2	2.9	3.8	3.5	5.7	14.9	13–19	pps0381	0.22	0.67
			qDM- Severe_non-4	4	2.9	3.8	4.9	4.8	13.5	15–25	pps0040	–0.74	–0.31
	Rehydration	Control	qDM-Post_irr-2	2	2.8	3.8	–	4.4	9.7	12–20	pps0381	0.56	1.01
			qDM-Post_irr-7	7	2.8	3.8	3.4	3.8	9.2	31–43	pps0705	0.64	–0.48
		Drought	qDM-Post_non-2.1	2	2.8	3.8	2.8	4.3	10.4	12–19	pps0395	0.39	0.61
			qDM-Post_non-2.2	2	2.8	3.8	–	3.9	13.5	47–68	pps0188	–0.40	–0.65
			qDM-Post_non-4	4	2.9	3.8	2.9	4.3	11.9	9–21	lpssr006	–0.79	–0.07
			qDM-Post_non-7	7	3.0	3.8	3.5	4.2	10.4	32–42	pps0705	0.53	0.15
LER	Severe	Control	qLER_irr-5	5	2.6	3.7	2.8	4.3	17.9	9–34	pps0857	0.11	0.33
LR	Severe	Control	qTS_irr-2	2	2.7	3.7	3.1	4.6	13.4	12–21	pps0523	–0.07	0.72
		Drought	qTS_non-2	2	2.7	3.8	2.7	3.8	10.3	12–20	pps0381	–0.36	0.45
			qTS_non-4	4	2.8	3.8	2.8	4.3	8.3	15–25	pps0040	–0.98	0.01

^a^ irr, control; non, drought treatment.^ b^ QTL nomenclature consists of the trait abbreviation followed by treatment and LG. LOD, logarithm of the odds ratio; LOD threshold, logarithm of the odds score for declaring significant QTL at *P* < 0.05; PV, phenotypic variation explained by QTL; 2-LOD interval, 2-LOD score support interval for QTL position; S, substitution effect of alleles from maternal parent; M, substitution effect of alleles from paternal parent. Sign indicates direction of effect, number indicates magnitude of effect.

A region at the proximal end of LG2 contains QTLs for shoot DM production at all four measurement time points and in both irrigated and non-irrigated treatments. This region also contains QTLs for LR under both treatments. A QTL region centrally located on LG7 was detected under irrigated and non-irrigated treatments but at the DM-Post measurement only.

By contrast, a region at the proximal end of LG4 contains QTLs specific to DM and LR under the non-irrigated treatment and only for DM production measured during severe drought (DM-Severe) and rehydration (DM-Post). The magnitude and phase of allelic effects (Table 7) at this QTL, which express the change in phenotype due to substitution of one parental allele for the other, indicated that the QTL effects are largely driven by alternative alleles segregating from the ‘Grasslands Samson’ parent. In this QTL region, genetic variation linked to alleles from the Moroccan parent was significant at DM-Severe, but was of lesser impact than ‘Grasslands Samson’, and was negligible for both DM-Post and LR (Table 7). By contrast, substantial allelic contributions from the Moroccan parent were indicated at other QTLs, most notably the LG2 QTL which was significant for growth under both treatments (Table 7).

QTL positions were evaluated for co-linearity with QTLs identified in other forage grasses and the model grass species rice, using the rice genome sequence as a template for comparison. Markers in the region associated with the LG4 QTL in perennial ryegrass (pps0040 and lpssr006) were aligned by *in silico* comparative analysis to a genome segment between 6–10Mb on rice chromosome 3 (Jones *et al.*, 2002*b*; Sim *et al.*, 2005). A total of 16 rice QTLs for traits related to drought response, from nine independent studies, align to this region (www.gramene.org). A QTL for tiller survival and herbage FW during severe drought detected in meadow fescue (*Festuca glaucescens*) is also in close proximity to this region of the rice genome (Alm *et al.*, 2011), based on the relative position of flanking marker CDO1395 (6.5Mb rice chromosome 3).

## Discussion

Defoliation of perennial ryegrass is essential to sustainable farm management practices (Parsons and Chapman, 2000), not only to feed animals but to avoid a drop in forage quality resulting from the rapid tissue turnover in this species (Carter, 1978; Acharya *et al.*, 2004). Volaire *et al.* (1998) suggested that the main developmental factor associated with persistence of perennial forage grasses in the field was the rate of herbage regrowth following recovery from dehydration and that this was closely correlated with tiller survival. In this study we have demonstrated that leaf lamina regrowth after defoliation was the most significant trait contributing to DM production throughout the non-irrigated treatment and after rehydration. The turgor pressure of enlarging cells has a significant impact on leaf lamina regrowth (Matyssek *et al.*, 1988). This is one of the first processes affected by, and most sensitive to, moisture deficit (Wardlaw, 1969; Hsiao, 1973; Bacon *et al.*, 1997). The positive correlation between DM production during severe drought and both Pn and RWC measured in the plant subset, as well as the negative correlation with δ13C, which provides an estimate of water use efficiency (Seibt *et al.*, 2008), leads us to suggest that the best performing plants were mainly reliant on dehydration avoidance mechanisms.

We have identified two main QTLs associated with areas of the genome that either support maintenance of shoot growth through the moisture deficit treatment or ensure the rapid recovery of growth after rehydration. The QTL on LG2 occurred in both stressed and non-stressed plants indicating an intrinsic involvement in plant vigour whilst the other, on LG4, is only evident under drought stress conditions and is associated with a drought adaptive region of the genome. The substantial contribution of alleles segregating from the ‘Grassland Samson’ parent in the LG4 QTL region suggests genetic variation for enhanced forage production and survival during drought already exists within New Zealand-adapted germplasm. This variation could be readily exploited, through applying a combination of selection following this novel drought protocol and the application of MAS, to produce new cultivars through conventional plant breeding. The potential significance of this area of the genome is supported by research in a related perennial forage grass, meadow fescue, where a QTL for tiller survival and herbage FW during severe drought was also identified at an aligned position (Alm *et al.*, 2011). Close correspondence between the ryegrass and meadow fescue moisture deficit response QTL on LG4 suggests that this area of the genome contains a gene, or genes, responsible for controlling a drought-responsive mechanism that is conserved across grass genera. Further to this, the meadow fescue QTL-flanking marker CDO1395 is linked to QTL for root length under stress in rice (Nguyen *et al.*, 2003). These kind of comparative genomic analyses may help inform improvements in other grasses and cereal crops of the *Poaceae*, as well as generate information to support fine mapping (Juenger *et al.*, 2005) and the identification and cloning of the underlying gene(s) in perennial ryegrass (Armstead *et al.*, 2008; Chen *et al.*, 2011; Sayed *et al.*, 2012; Shinozuka *et al.*, 2011).

Perennial ryegrass cultivars that have a propensity for greater spring growth are considered desirable (Kennedy and O’Donovan, 2013). This is mainly because growth conditions during the spring are usually optimal for the plants, water availability is not limiting, and the plants are able to respond to these conditions (Parsons and Chapman, 2000). Consequently, spring DM production has been targeted through selective breeding for plant improvement in pasture-based farming systems to support increased animal production (Burke *et al.*, 2007; Kennedy and O’Donovan, 2013). We have shown here that early plant vigour in the spring, measured as DM-Pre, was associated with increased DM production in the initial phase of the non-irrigated treatment. However, caution is advised as this trait was not found to be associated with DM production under severe drought conditions and during rehydration.

DM production of the irrigated clones was found to be the second most significant trait explaining improved DM production of the clones under moisture deficit and during rehydration. However, this measure explained only 8% of the variation in DM production measured during the severe drought stage and 12% of the variation after rehydration. This suggests that genotype vigour, estimated here by the DM production of the irrigated clones at each time point, which is dependent on both plant adaptation traits and genotype × environment interactions (Conaghan *et al.*, 2008), also has an influence on plant performance when under moisture stress. Significantly, this also suggests that selecting for moisture deficit tolerant plants does not necessarily result in lower DM production under non-limiting water conditions.

The occurrence of QTLs for DM and LR at the same LG2 position in both irrigated and non-irrigated treatments and at all of the time points throughout the treatment schedule suggests that in this experiment we have identified a region of the genome that contains a gene, or genes, that support shoot growth and plant vigour, independent of the environmental conditions. The introgression of exotic alleles associated with plant persistence, nutritive value, and nutrient and water use efficiency into adapted material has previously been demonstrated to be successful in wheat, rice, tomato, and maize (Tanksley and Nelson, 1996; Wang and Chee, 2010). The influence of alleles sourced from the Moroccan parent in the LG2 QTL region in the current study highlights the potential contribution of alleles from ‘exotic’ germplasm for the improvement of agronomically significant traits in forage grasses. The alignment of QTL position and allelic effects for both DM and LR, at both the LG2 and LG4 positions, indicates that parallel selection for these traits is feasible and is suggestive of a pleiotropic effect at the respective loci. This is a significant finding as the pastoral industries are currently lacking productive perennial grass species that can tolerate defoliation and moisture deficit (Dear and Ewing, 2008), and may have broader implications for the improvement of other perennial clonal crop species.

The model used in this study captures a significant proportion of the variation in DM production through the course of the experiment. However, 55%, 35%, and 45% of the variation at the mid drought, severe drought and rehydration stages, respectively, remains unexplained. Genotypic variation in other traits associated, for example, with nutrient uptake or metabolite accumulation might explain a portion of this ‘missing’ variation. The uptake and acropetal translocation of nutrients can be impaired when plants are exposed to moisture stress (Hu and Schmidhalter, 2005). Perennial ryegrass plants rely on other factors, including the accumulation of water soluble carbohydrates, for superior survival during moisture stress, particularly after defoliation when the photosynthetic capacity of the plant is reduced (Parsons *et al.*, 1988; Volaire *et al.*, 1998). Busso *et al.* (1990) showed that perennial ryegrass plants exposed to a prolonged period of moisture deficit plus defoliation could have rapid initial regrowth because of the high amounts of water-soluble carbohydrate accumulated in their storage organs during stress. It is also possible that other metabolites could contribute to the survival and enhanced performance of some of the genotypes and greater understanding will result from further studies on the metabolite composition of contrasting material carried out in the future.

There is increasing evidence in the literature that many species, including some grasses, can passively relocate water from the soil through hydraulic redistribution (Caldwell *et al.*, 1998; Leffler *et al.*, 2005; Howard *et al.*, 2009). Other factors, such as the type and composition of the soil (Kachanoski *et al.*, 1988), difference in soil compaction (Chesson and Warner, 1981; Guo *et al.*, 2002; James *et al.*, 2003) and variation produced by irrigation systems (Tarjuelo *et al.*, 1999) are also known to impact on SWC homogeneity. However, even though considerable effort was put into minimising SWC variation created by the above factors and any variation that occurred was measured precisely in this experiment, we could not detect any evidence of a genotype effect on SWC.

In summary, the protocol developed in this study has proven to be an efficient and accurate method for screening populations and identifying individual genotype responses to abiotic stress. The application of this protocol promises to support the identification and selection of plant genotypes with enhanced growth potential under moisture deficit and to differentiate drought specific responses from general plant vigour-related growth.

## Funding

This study was supported by AgResearch investment via the Research and Capability fund.

## Abbreviations:

BLAT: BLAST-like alignment Tool
BLASTN: nucleotide basic local alignment search tool
CP: cross pollination
DM-Pre: dry matter yield before initiation of the drought treatment
DM-Mid: dry matter yield at mid drought
DM-Severe: dry matter yield at severe drought
DM-Post: dry matter yield at rehydration stage
ILGI: International *Lolium* Genome Initiative
IM: interval mapping
LER: leaf elongation rate
LOD: logarithm-of-odds score
MAS: marker-assisted selection
MQM: multiple QTL mapping
PV: phenotypic variance
QTL(s): quantitative trait locus (loci)
SSR: simple sequence repeat
SWC: soil water content
LR: leaf lamina regrowth after defoliation.
